# Advancements in Detoxification of Municipal Solid Waste Incineration Fly Ash: A Review of Hazardous Properties, Treatment Strategies, and Resource Utilization

**DOI:** 10.3390/ma19061157

**Published:** 2026-03-16

**Authors:** Kun Li, Jixin Deng, Junjie Zhang, Hanlin Shen, Bo Liu

**Affiliations:** 1 Institute for Advanced Materials and Technology, University of Science and Technology Beijing, Beijing 100083, China; 2 Shunde Innovation School, University of Science and Technology Beijing, Foshan 528399, China

**Keywords:** MSWI fly ash, heavy metals, detoxification, resource utilization, thermal treatment

## Abstract

Municipal solid waste incineration (MSWI) fly ash is classified as hazardous waste due to its enrichment of heavy metals and dioxins. This article systematically reviews its generation pathways, physicochemical characteristics, and potential environmental risks, based on the literature from 2010 to 2025 sourced from Web of Science, Scopus, ScienceDirect and China National Knowledge Infrastructure. Emphasis is placed on heavy metal stabilization, dioxin degradation and resource recovery from MSWI fly ash. The mechanisms, technical advantages, and application limitations of three mainstream detoxification, including solidification/stabilization, extraction and thermal treatment, were emphasized. For instance, geopolymer achieves >99.6% Pb immobilization and electrodialytic removal rates of Cd up to 98%, while vitrification reduces the MSWI fly ash volume by >50%. A comprehensive exploration of MSWI fly ash resource utilization was conducted, covering the preparation of ceramic tiles, synthesis of glass ceramic and glass ceramic foams, processing of road substrates, and modification of cement-based composite materials. The current technological system still faces challenges such as high costs, excessive energy consumption, and secondary pollution. Future research should focus on developing green, low-carbon, and low-cost processes, improving long-term environmental stability of products and strengthening pollution source reduction control.

## 1. Introduction

Rapid economic development and improvements in living standards have contributed to a substantial rise in municipal solid waste (MSW), presenting a significant challenge to urban management. World Bank estimates show that global urban areas will produce 3.4 billion tons of MSW by 2050 [1,2], with China and the European Union (EU) generating an annual generation of 230 and 250 million tons each. MSW is disposed of mostly by landfilling and incineration. Due to increasing constraints on land availability and environmental resources, the landfill is progressively being phased out [3]. Municipal solid waste incineration (MSWI) is a viable alternative, enabling thermochemical conversion of waste to reduce volume by up to 90% and mass by approximately 70% [4], aligning with the modern waste management principles of harm reduction, volume minimization, and resource recovery, with additional advantages such as elimination of pre-treatment, high processing efficiency, rapid treatment, and waste heat utilization [1,5].

However, MSWI produces hazardous MSWI fly ash; its disposal and resource utilization has become an urgent problem that hinders the promotion and application of MSWI [6]. While several reviews have addressed MSWI fly ash management, they often focus on a single technology stream or lack integrated analysis across the entire detoxification-to-resource chain.

This paper analyzes the production mechanism of MSWI fly ash and the sources and hazards of heavy metals and dioxins, summarizes the effective ways of harmless disposal and resource utilization methods in recent years, analyzes the mechanisms, advantages and disadvantages of MSWI fly ash treatment, and discusses the ways to further improve its application effect. The aim is to provide some constructive suggestions for further MSWI fly ash treatment, offer data and theoretical support for the formulation of low-consumption and low-carbon management policies, and point out the core direction for the research and development of new treatment technologies.

## 2. Review Methodology

To align with the aforementioned objectives, a structured, multi-step methodology was defined to ensure the reproducibility of the literature selection process and the robustness of the subsequent data analysis.

A search strategy was developed across Web of Science, Scopus, ScienceDirect and China National Knowledge Infrastructure using keywords, including “MSWI fly ash”, “municipal solid waste incineration bottom ash”, “incineration residues”, “immobilization”, “solidification/stabilization”, “thermal treatment”, “chemical extraction”, “geopolymer”, “resource utilization”, “valorization”, “recycling”, “life cycle assessment”, “leachability assessment”. The initial search was restricted to peer-reviewed journal articles, review papers, and book chapters published between 2010 and 2025.

**Figure 1:**
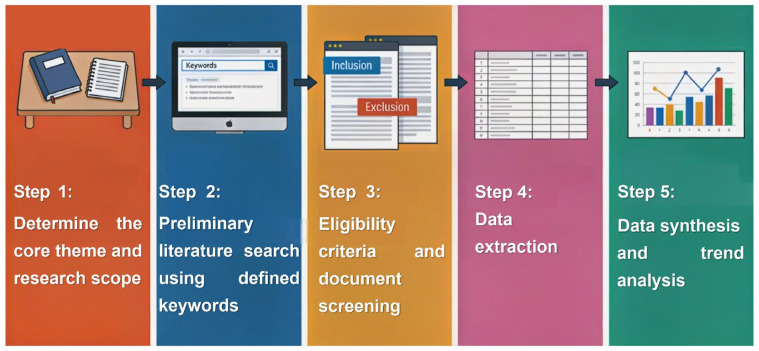
Flowchart of the review methodology.

In order to filter the relevance and quality of the retrieved documents, a two-stage screening process was implemented, including title/abstract screening, followed by full-text screening, and selection was made based on compatibility with the article. The flowchart of this review methodology is shown in Figure 1. The specific steps can be briefly summarized as follows: (1) determine the core theme and research scope, (2) perform a preliminary literature search using defined keywords, (3) perform eligibility criteria and document screening, (4) perform data extraction and (5) perform data synthesis and trend analysis.

## 3. Characteristics and Environmental Impact of MSWI Fly Ash

### 3.1. Generation and Characteristics of MSWI Fly Ash

**Figure 2:**
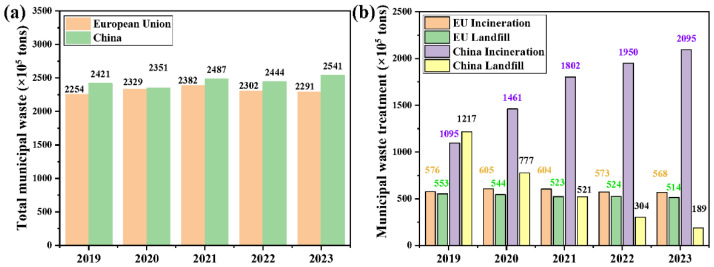
Comparison of municipal solid waste between the European Union and China: generation (**a**) and treatment (**b**) [7,8]. The incineration treatment indicates incineration with energy recovery, and European Union (EU) includes 27 countries from 1 February 2020.

As illustrated in Figure 2a, data from 2019 to 2023 indicates that both EU and China face significant challenges in managing large volumes of MSW [7,8].

However, substantial differences exist in EU’s and China’s waste disposal strategies. Supported by the legislative effort for “zero-waste cities”, China has significantly expanded its MSWI capacity, with the amount of incinerated MSW increasing from approximately 109.5 million tons in 2019 to 209.5 million tons in 2023 (Figure 2b). During the same period, landfilling decreased from 121.7 million tons to 18.9 million tons (Figure 2b). Notably, the total MSW generation in China and the EU is comparable, yet China’s population is approximately three times that of the EU, meaning the per capita MSW generation in the EU is significantly higher than in China. This difference is attributed to variations in consumption patterns, urban living standards, and waste sorting systems between the two regions. In addition, the EU has a more mature and diversified waste disposal system with high recycling, while China’s MSW disposal is currently dominated by incineration and landfilling.

In contrast, the EU’s MSW disposal structure has remained relatively stable over the past five years. Landfilling has remained at around 51–52 million tons per year, while incineration has also fluctuated narrowly between 54 and 60 million tons, with no significant trend toward structural change observed. This indicates that the EU’s waste management system has entered a mature phase, characterized by slower capacity expansion and more gradual optimization of treatment processes.

**Figure 3:**
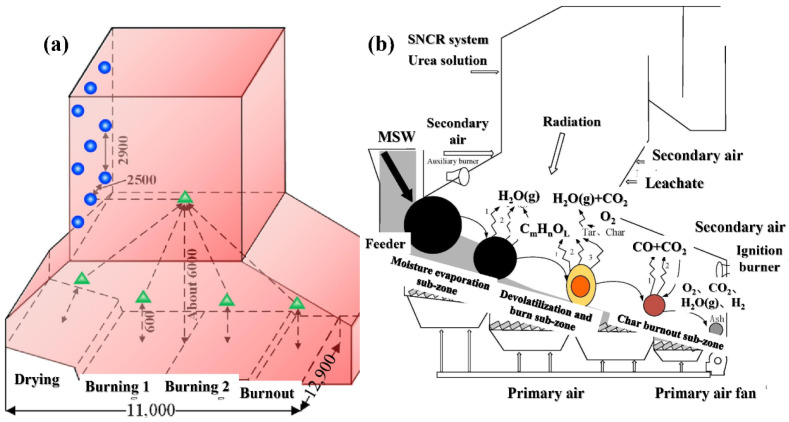
(**a**,**b**) Mechanical grate furnace [10,11].

The mechanical grate furnace is currently the most widely used in the world, accounting for about 80% of the MSWI market [9]. As shown in Figure 3a,b, it mainly agitates the MSW through the mechanical movement of the grate, promoting the complete combustion of the MSW.

The temperature of MSWI in the furnace is higher than 850 °C, while the temperature of the gas can exceed 1200 °C. MSWI fly ash is the residue trapped by heated flue gas purification during the above mechanical grate furnace incineration or fluidized bed incineration [12]. Typically, MSWI fly ash is about 3% to 5% of the amount of MSW disposal capacity [13]. MSWI fly ash has high leaching concentrations of heavy metals and high toxic equivalents of substances such as dioxins; it is therefore included in the National Hazardous Waste List. At present, many countries have implemented policies to control and manage MSWI fly ash, as shown in Table 1 [14].

MSWI fly ash is captured by air pollution control device systems, whose core units (bag filters, lime injection, activated carbon adsorption) directly shape ash physicochemical properties. Bag filters retain fine particles (<50 μm) with a high specific surface area (170–1000 m^2^/kg); lime injection neutralizes acid gases, raising ash pH to 8–12 and increasing chloride content; activated carbon efficiently adsorbs PCDD/Fs (reducing concentrations to <0.1 ng I-TEQ/g) but introduces carbon impurities. Regional air pollution control device configurations lead to distinct ash properties, which further determine the selection and efficiency of subsequent detoxification and resource utilization technologies [15]. The main constituents of MSWI fly ash include inorganic salts, heavy metals, and dioxin-related compounds. Its physical and chemical properties are determined by the incineration technology employed, operational conditions and the composition of MSW [14]. Generally, the phases of MSWI fly ash contain CaCO_3_, CaCl(OH), NaCl, KCl, CaSO_4_, and various silicates (e.g., quartz) [16].

### 3.2. Heavy Metals in MSWI Fly Ash

Heavy metals generally refer to metals with densities greater than 5 g/cm^3^ in nature [17]. The types and concentration of heavy metals in MSWI fly ash are related to the types of MSW incinerated, as summarized in Table 2. The predominant heavy metals present in MSWI fly ash include Cr, Cu, Ni, Pb, Zn, Cd, and Hg, with Zn and Pb consistently exhibiting the highest concentrations, reaching 3317 mg/kg and 486 mg/kg in China, respectively [18,19].

Heavy metals undergo migration and partition among bottom ash, fly ash, and other by-products. This distribution behavior is governed by complex mechanisms, including evaporation–condensation, mechanical entrainment, and adsorption onto fly ash particle. The distribution of heavy metals during MSWI is largely governed by their volatility [21]. Those with high vapor pressure and low boiling points tend to volatilize under incineration conditions and subsequently transition to the gas phase, whereas metals characterized by low vapor pressure and high boiling points predominantly remain in the bottom ash [22].

Chlorine and sulfur in MSW significantly influence heavy metal speciation and distribution during incineration. This effect arises from the formation of metal chlorides and sulfates under high-temperature conditions. Since most heavy metal chlorides exhibit lower boiling points than their elemental counterparts, they demonstrate a greater tendency to volatilize, thereby altering the overall partitioning behavior of heavy metals in the process. Other forms of substances will also be formed during the MSWI process; ultimately, heavy metals will exist in MSWI fly ash in the form of elemental substances, oxides, chlorides, sulfates, carbonates, phosphates, and silicates (Table 2) [20].

Under conditions of elevated heavy metal content, MSWI fly ash exhibits significant leaching potential, particularly in acidic environments. Following leaching, heavy metals can migrate into groundwater, soil, and the atmosphere, posing direct risks to human health or indirectly through bioaccumulation within food chains [23]. The leaching behavior of heavy metals from MSWI fly ash is influenced by several factors, including the intrinsic physicochemical properties of the ash, liquid-to-solid ratio, and pH. For instance, a decrease in particle size, which corresponds to an increase in specific surface area, accelerates the leaching kinetics of heavy metals. This enhanced reactivity may thereby facilitate the release and mobilization of metallic contaminants into the environment [24,25]. The characteristics and toxicity of heavy metals differ with existence forms. Most heavy metals have complicated transmission routes, which are difficult to decompose and easy to accumulate [14].

### 3.3. Dioxins in MSWI Fly Ash

MSWI continues to expand worldwide. However, this development is often hampered by the “Not in My Back Yard” phenomenon, largely driven by public concern over the formation of highly toxic dioxins during the incineration process. MSWI fly ash can contain dioxin concentrations as high as 5 ng/g. With a toxicity approximately 900 times that of arsenic and 1000 times that of potassium cyanide, dioxins are regarded as the most toxic organic compounds known to science [26].

**Figure 4:**
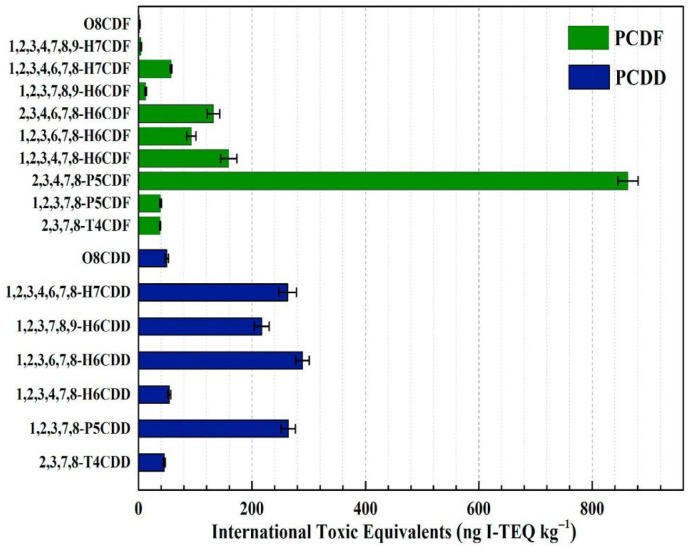
International toxic equivalents of 17 toxic PCDD/Fs in the MSWI fly ash [32].

Dioxins represent a class of chlorinated organic compounds encompassing poly-chlorinated dibenzo-p-dioxins (PCDDs), poly-chlorinated dibenzofurans (PCDFs), and their related congeners. These compounds comprise eight homologous groups, with 75 PCDD and 135 PCDF isomers arising from variations in the number and position of chlorine substituents. Among these, 17 PCDD/Fs are considered particularly toxic and are commonly found in MSWI fly ash. The international toxic equivalency values for these 17 toxic congeners are summarized in Figure 4.

Dioxins are colorless and odorless compounds that exist primarily in gaseous and solid forms. During the process of MSWI, they are predominantly present as particulate matter in the flue gas or adsorbed onto the surface of fly ash particles. These compounds exhibit high molecular symmetry, which underpins their remarkable chemical stability under acidic, alkaline, and redox conditions. Dioxins are practically insoluble in water but readily soluble in most organic solvents and exhibit high lipophilicity. Their low vapor pressure contributes to limited volatility in the environment. Owing to their persistence and bioaccumulation potential, dioxins accumulate in living organisms and exert long-term adverse effects on ecosystems and human health [27,28]. The International Cancer Center has listed it as a primary carcinogen due to its reproductive toxicity [29].

Dioxins primarily form within the temperature range of 200–650 °C and can be effectively decomposed when the combustion temperature exceeds 800 °C [30]. The recognized dioxin formation pathway can be roughly divided into (1) inherent in MSW; (2) high-temperature homogeneous pathway; (3) low-temperature heterogeneous pathway [31].

(1) Inherent dioxins in MSW

MSW inherently contains trace amounts of dioxins and these pre-existing compounds are not considered the primary source of dioxin emissions from incineration. This is because, upon entering the incinerator, MSW is subjected to temperatures typically maintained above 850 °C, with flue gas residence times exceeding 2 s and more than 99.95% of dioxins being effectively decomposed. Beyond temperature and residence time, excess oxygen levels affect the oxidation state of metals and dioxin formation; typically, >6% O_2_ is recommended to suppress PCDD/Fs synthesis. Turbulence and mixing efficiency determine the completeness of combustion and the entrainment of particulates [33]. Thermal gradients and cooling rates in the post-combustion zone critically impact dioxin reformation; rapid quenching (>100 °C/s) from 400 °C to 200 °C can reduce PCDD/F formation by >90% [10]. Therefore, optimization of combustion conditions can facilitate the decomposition of dioxins carried by MSW.

(2) High-temperature homogeneous pathway

**Figure 5:**
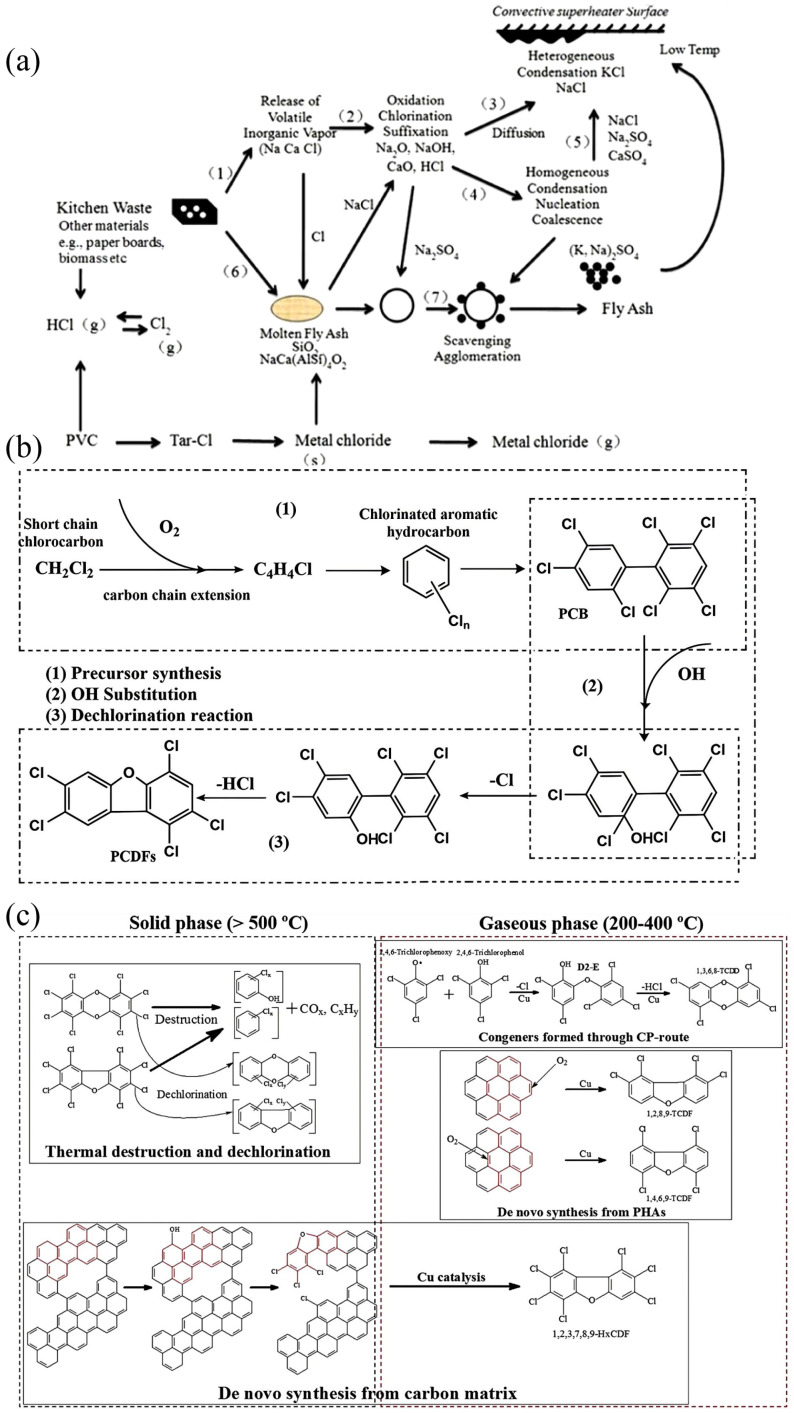
(**a**) Migration path of chlorine during MSW combustion [34], (**b**) pathway of high-temperature gas phase reaction for PCDFs, (**c**) transformation pathways of PCDD/Fs during thermal treatment of MSWI fly ash [35].

The charging of substantial waste feedstock can locally restrict air circulation and create oxygen-deficient conditions. This results in incomplete combustion, thereby promoting the formation of PIC (products of incomplete combustion), with the associated gas phase synthesis predominantly occurring in the high-temperature heat exchange zone of the incinerator [26]. During the incineration of MSW, the transformation and migration of chlorine are illustrated in Figure 5a. The majority of chlorine is released into the flue gas in the form of HCl, while a portion may react with oxygen to generate H_2_O and Cl_2_. A small fraction is also directly converted into Cl_2_. PIC often exhibit structural similarities to dioxins and can undergo chlorination to form chlorinated PIC. Aliphatic olefins or alkynes in PIC can generate chlorobenzene through chlorination reaction, and chlorobenzene undergoes hydrocarbon chlorination reaction with compounds containing hydroxyl groups to generate chlorobiphenyls. Finally, in the combustion zone at temperatures between 870 and 980 °C, chlorobiphenyls can act as precursors for the formation of PCDD/Fs [31]. The formation paths of PCDF high-temperature gas phase reaction are shown in Figure 5b [26].

(3) Low-temperature heterogeneous pathway

The flue gas produced by MSWI contains fatty compounds (ethylene and acetylene), as well as aromatic organic compounds (chlorobenzene), and may also contain PCDD/Fs generated by high-temperature gas reaction. In addition, it contains the incomplete combustion of carbon and partial transition metals (Cu, Fe, etc.) due to local hypoxia. After the flue gas passes through the high-temperature section, the temperature decreases below 850 °C. These substances will undergo a polymerization reaction in the low-temperature section and then generate dioxins again. This is the heterogeneous catalytic generation at low temperature. Consequently, this stage is responsible for generating the majority (>50%) of dioxins. A comprehensive overview of the transformation pathways of PCDD/Fs across the entire thermal treatment process is provided in Figure 5c.

## 4. Harmless Disposal of MSWI Fly Ash

The harmless disposal of MSWI fly ash entails the immobilization or removal of heavy metals and dioxins to a substantial extent via physiochemical processes. The treated residue must comply with regulatory standards for subsequent utilization or secure landfilling. A sustainable development strategy necessitates that harmless disposal techniques for MSWI fly ash address economic, environmental, and safety considerations concurrently. The technologies currently prevalent on a global scale mainly encompass solidification/stabilization, extraction, and thermal treatment [36].

### 4.1. Solidification/Stabilization

As a primary treatment for MSWI fly ash, S/S encompasses two key mechanisms: solidification, which involves the addition of additives to physically encapsulate hazardous components within a solid matrix, and stabilization, wherein additives chemically react with the ash to convert harmful substances into less soluble and low-toxicity forms [37]. The main technologies include cement solidification, geopolymer solidification, chemical solidification/stabilization.

#### 4.1.1. Cement Solidification

Cement S/S technology is widely recognized as one of the most effective for mitigating the adverse effects of MSWI fly ash on both human health and the ecological environment [38]. This recognition stems primarily from its cost-efficient operation and the well-documented long-term stability of the resulting solidified matrix.

The process initiates with the formation of primary hydration products, namely C-S-H, C-A-H, and calcium hydroxide. Following this, OH^−^ and Ca^2+^ ions derived from calcium hydroxide decomposition further react to yield insoluble C-S-H and C-A-H gels [39]. Upon the incorporation of MSWI fly ash in a designed cement mixture, hazardous components including heavy metals and dioxins are physically encapsulated and chemically bound within the developing gel matrix, which significantly reduces their leaching potential.

However, the mass after cement solidification increases dramatically, making it more difficult to transport materials to landfills [40]. In addition, the addition of MSWI fly ash will reduce the compressive strength of cement products [41], and the aging and carbonization of the prepared products will also increase calcium carbonate in the pores. Once the limit value is exceeded, additional internal pressure and cracks will be generated, once again reducing the strength of the product [42].

Water washing can be used as a pre-treatment step for cement solidification to remove chlorine elements from the raw materials. However, water washing can lead to a lack of Ca in the raw materials, which affects the effectiveness of cement solidification. Chen et al. [43] developed a multi-step circulating water washing method that can improve the removal rate of chlorine from MSWI fly ash without affecting other soluble substances.

#### 4.1.2. Geopolymer Solidification

Cement production is energy-intensive, consuming substantial fossil fuels and releasing pollutants (CO_2_, CO, NO_x_, SO_2_, and particulate matter) [44]. According to statistics, the cement production industry accounts for 5–8% of man-made CO_2_ emissions, making it a major contributor to global warming [45,46]. Geopolymers derived from MSWI fly ash represent a promising alternative to conventional cement, mitigating its environmental footprint [47].

**Figure 6:**
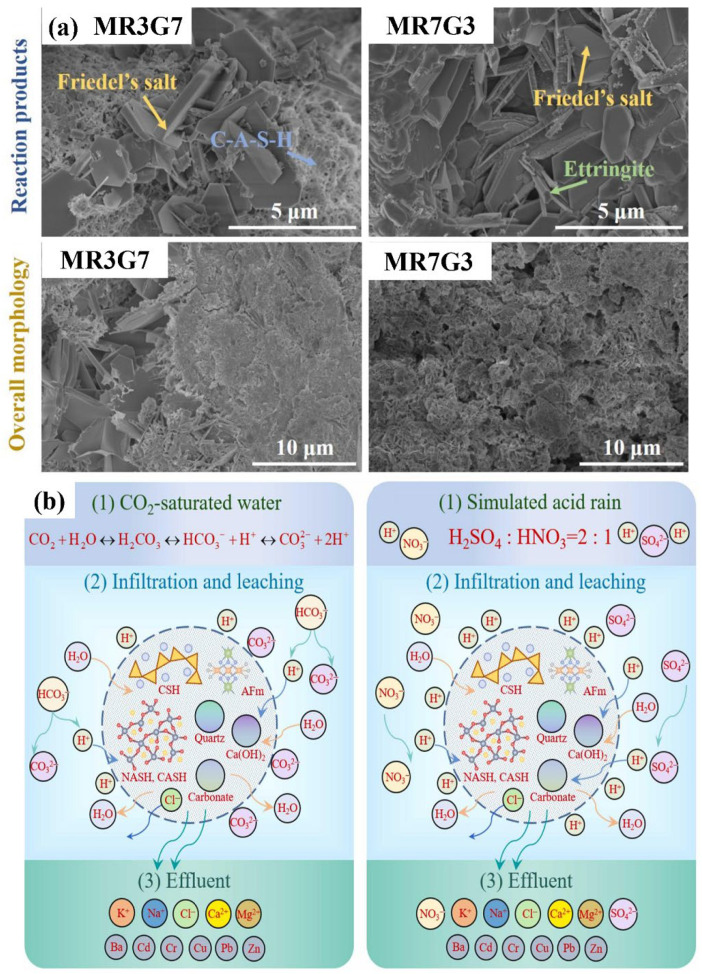
(**a**) SEM of geopolymer samples with different content of MSWI fly ash [49]; (**b**) dissolution mechanism of heavy metals in geopolymer or cement under different corrosion conditions [56].

Geopolymer solidification involves the combination of alkaline activator solution and aluminosilicate precursor to form inorganic polymer, and then the obtaining of specific curing [48]. The mechanical strength of the resulting geopolymer is largely determined by the properties of the precursor [48]. MSWI fly ash served as both a precursor and a source of alkalinity, offering the dual advantage of reducing production costs and promoting waste utilization when incorporated into the mixture. However, increasing the proportion of MSWI fly ash has been found to delay the early-stage reaction kinetics of geopolymerization, posing a challenge to processing efficiency. Liu et al. [49] introduced an innovative method for developing high-performance one-part geopolymers by synergistically employing MSWI fly ash and red mud (RM) as composite alkali activators. The incorporation of RM was found to significantly enhance early reaction kinetics, promoting the formation of amorphous C-A-S-H and crystalline phases, including ettringite and Friedel’s salt (Figure 6a). With an optimal MSWI fly ash-to-RM ratio of 3:1, the synthesized geopolymer achieved a 28-day compressive strength of 57.6 MPa, representing a 138% improvement over systems using MSWI fly ash alone. Furthermore, the reaction products effectively immobilized potentially toxic elements and chlorides through combined physical encapsulation and chemical stabilization, demonstrating a Pb immobilization efficiency exceeding 99.6%.

However, increasing the amount of MSWI fly ash will also lead to an increase in chlorine and sulfate, which in turn has an adverse effect on the durability of geopolymers. Therefore, MSWI fly ash can be pretreated by water washing to remove chlorine and sulfate, reduce environmental risks, and enhance the performance of the geopolymer [50]. However, the waste water requires further treatment (e.g., precipitation, reverse osmosis) before discharge.

Heavy metal ions in geopolymer can be immobilized within the geopolymer through multiple mechanisms: incorporation into the aluminosilicate network, charge balancing within the framework, and precipitation as insoluble compounds [51]. Zheng et al. [52] employed the partial charge model to analyze the heavy metal ion reactions involved in the geological polymerization process in MSWI fly ash. The research shows that heavy metal ions can undergo a condensation reaction with aluminosilicate and aluminate components can promote the condensation of heavy metal ions. However, considering that heavy metal cations can also be used as the formers or modifiers of geopolymer networks, as well as in atomic coordination in the framework, the fixation effect of this mechanism on heavy metal cations cannot be excluded at present. Su et al. [53] found that Pb^2+^ and Cd^2+^ are effectively immobilized in the aluminosilicate gel structure through substituting the Ca^2+^ in the C-S-H and C-A-S-H phases (Equations (1) and (2)).

(1) $$C\text{-}S\text{-}H+{Pb}^{2+}({Cd}^{2+})\to C\text{-}S\text{-}H\text{-}Pb(Cd)+{Ca}^{2+}$$

(2) $$C\text{-}A\text{-}S\text{-}H+{Pb}^{2+}({Cd}^{2+})\to C\text{-}A\text{-}S\text{-}H\text{-}Pb(Cd)+{Ca}^{2+}$$

Based on a comparative analysis of heavy metal immobilization in MSWI fly ash, Fan et al. [54] demonstrated that geopolymers exhibit superior performance over Portland cement. This enhanced retention is attributed to the participation of active aluminosilicates during the geopolymerization process, leading to the formation of new phases such as Friedel’s salt and brucite. These phases contribute to a more compact microstructure and improved mechanical properties of the final matrix [55]. Liang et al. [56] identified that the long-term leaching of heavy metals follows a two-stage leaching pattern (rapid release followed by gradual stabilization), noting a lower cumulative release from geopolymers than from Portland cement. They attributed this to two main dissolution mechanisms (Figure 6b): (1) corrosion/replacement of H^+^ from the acidic simulated acid rain, weakening the stability of substances that immobilize heavy metals; (2) counter-ion effect, where anions like SO_4_^2−^, NO_3_^−^, Cl^−^ promote heavy metal release via chemical complexation or electrostatic interactions.

However, the widespread application of MSWI fly ash-based geopolymers is hindered by the variability in ash composition, which stems from regional and socio-economic factors, resulting in unstable performance of the final product despite consistent synthesis conditions [51].

#### 4.1.3. Chemical Solidification/Stabilization

Chemical S/S is a technique that employs chemical stabilizers to react with toxic substances, primarily heavy metal ions, in MSWI fly ash. This reaction leads to the formation of insoluble precipitates or cross-linked stable complexes, thereby reducing their solubility and mobility. The chemical reagents used in this method can be divided into inorganic chemical reagents and organic chelating reagents [57]. Cement solidification is being replaced by chelating agent stabilization as MSWI fly ash production increases [58]. Ma et al. [59] evaluated the stabilization effect of heavy metals in MSWI fly ash using cement and four chemical chelating agents (dithiocarbamate, dithiocarbamic acid dipotassium salt, amino dithiocarbamate chelating resin, and thiourea). Their results demonstrated that dithiocarbamate exhibited optimal stabilization performance, which was linked to its three-dimensional complexation structure, whereas the other agents had a two-dimensional structure. With the addition of 2% and 3% (*w*/*w*) of dithiocarbamate, the leaching concentrations of Cd, Pb, and Ni were effectively suppressed below the regulatory limits.

Table 3 summarizes the effects of different chemical stabilizers on the leaching characteristics of heavy metals in MSWI fly ash under neutral and acidic conditions.

The chelating agent demonstrates pH-sensitive behavior, with the resultant solidified matrix exhibiting an exceptionally high specific surface area accompanied by a porous structure defect. Ensuring the long-term stability of chemically solidified/stabilized MSWI fly ash remains a critical challenge. The integrated approach of combining a chemical chelating agent with cement-based solidification has been proposed as a strategic supplement to ensure the heavy metal immobilization effect. This integrative methodology synergistically leverages the selective binding mechanisms of chelating agents with the physical encapsulation properties of cementitious materials, thereby establishing a dual protection barrier against heavy metal leaching. Chen et al. [60] conducted a systematic assessment of an integrative stabilization strategy combining cement solidification with NaH_2_PO_4_ for treating lead (Pb) in MSWI fly ash. The experimental findings demonstrate that while individual processes (20% cement or 5% NaH_2_PO_4_) reduced leachable Pb below the regulatory threshold of 0.25 mg/L for landfill disposal, the synergistic stabilization methodology (10% cement + 2% NaH_2_PO_4_) achieved superior immobilization, lowering Pb leaching to 0.005 mg/L and increasing the residual Pb fraction to 77.05%. Through response surface methodology, optimized parameters at 11.64% cement, 2.79% NaH_2_PO_4_, and a 0.48 liquid-to-solid ratio simultaneously minimize the volume expansion ratio and treatment cost (253 CNY/t). Du et al. [58] compared the long-term stability of MSWI fly ash stabilized by cement and chemical solidification/stabilization techniques under natural aging, and found that combining chemical stabilization and cement solidification increased the long-term stability of heavy metals and reduced their environmental risks.

#### 4.1.4. Analysis and Comparison of Solidification/Stabilization Technologies

While S/S technologies effectively reduce immediate leaching risks, their long-term efficacy and sustainability vary significantly. Cement solidification remains the industrial benchmark due to its operational simplicity and predictable performance, yet it suffers from high carbon footprint (~0.85–0.95 t CO_2_/t cement, mainly from clinker production), with a 20–30% volume increase, as well as susceptibility to acid attack, leading to long-term metal remobilization. Geopolymer solidification reduces CO_2_ emissions by 40–60% due to the avoidance of calcination and superior mechanical and immobilization properties, as shown in Table 4.

However, its sensitivity to precursor composition, especially Cl and sulfate content and the cost/alkalinity of activators, hinder standardization and scale-up. Chemical chelation provides rapid, high-efficiency stabilization for specific metals but raises concerns about the long-term stability of organic–metal complexes and potential secondary pollution. An integrated approach, such as low-dose chelation followed by geopolymer encapsulation, appears promising for balancing cost, performance, and durability, but requires more life cycle assessment studies.

Table 4 shows the summary of the solidification/stabilization style and efficiency.

### 4.2. Extraction

Extraction technologies leverage the distinct physicochemical properties of MSWI fly ash components to separate and recover heavy metals via chemical, biological, or electrodialytic approaches [68]. In this process, heavy metals are first transferred into a leaching solution, from which they are subsequently extracted for resource recovery [69].

From a long-term perspective, extractive methodologies demonstrate optimal leaching efficacy in the environmentally sound management of MSWI fly ash, effectively mitigating long-term environmental risks associated with heavy metal contamination through permanent sequestration. This approach facilitates a dual strategy of environmental risk mitigation and resource recovery, as the mobilized heavy metals can undergo subsequent electrochemical recovery processes. Contemporary research identifies three predominant extraction techniques: electrodialysis-mediated separation, microbial-assisted bioleaching, and chelation-enhanced chemical extraction.

#### 4.2.1. Electrodialytic Extraction

**Figure 7:**
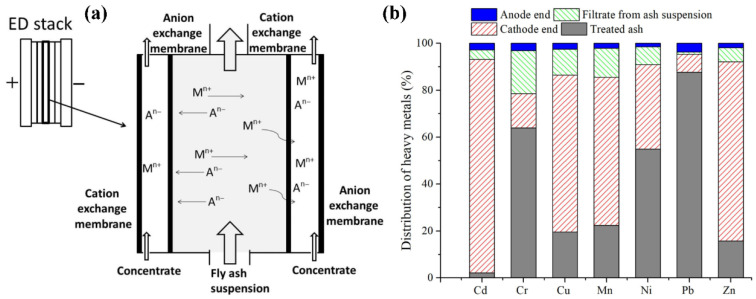
(**a**) Diagram of electrodialytic [71]; (**b**) distribution of heavy metals after electrodialytic [74].

Electrodialytic treatment of MSWI fly ash, comprising EDSE (electrodialytic separation) and EDR (electrodialytic remediation), is driven by a DC (direct current) field. In this process, MSWI fly ash or its leachate is strategically positioned within specialized electrochemical cells. Under a precisely modulated DC electric field, selective ion transport is achieved through the ion-exchange membranes, inducing the rapid electrophoretic movement of ionic species. This results in the efficient segregation of target heavy metals from the aqueous matrix via electrokinetic phase separation [70]. Heavy metal species in MSWI fly ash exhibit characteristically high ionic charge densities. When subjected to an applied current, these ions migrate from the MSWI fly ash into the cathode chamber by traversing a cation-exchange membrane, thereby achieving decontamination. The transfer mechanism schematic is shown in Figure 7a [71].

The treatment of MSWI fly ash leachate by electrodialytic is mainly embodied in two aspects: separation, concentration and recovery. EDSE can effectively remove Cu, Hg, Zn and other heavy metals, but has little effect on Cd, Cr and Pb removal. Nancharaiah [72] summarized the electrode potential required for the recovery of various heavy metals, and pointed out that the removal effect of various heavy metals is affected by pH, initial concentration of the leaching solution, electrode, membrane and other factors. And the effects on different heavy metals are different [73].

Zhan and Kirkelund [74] demonstrated distinct electromigration efficiencies for heavy metals in EDR of MSWI fly ash, with Cd^2+^ (98%), Cu^2+^ (80%), Mn^2+^ (78%), and Zn^2+^ (84%) exhibiting superior removal rates compared to Cr (36%), Ni (45%), and Pb (12%). This disparity is attributed to the effective migration and concentration of Cd^2+^, Cu^2+^, Mn^2+^, and Zn^2+^ at the cathode side of the electrodialysis cell, enabling their efficient removal, while other heavy metals were retained in the MSWI fly ash. Additionally, the removal of Pb was particularly hindered by the formation of low-solubility PbSO_4_ precipitates resulting from the reaction between Pb^2+^ and SO_4_^2−^. The distribution of heavy metals in EDR is shown in Figure 7b.

The removal rate of heavy metals by electrodialytic can be improved by changing the form of heavy metal ions in MSWI fly ash. The main methods include washing MSWI fly ash, reducing pH, changing redox conditions, etc. Chen et al. [75] obtained the maximum leaching amount of heavy metals studied in MSWI fly ash by combining water washing, electrodialytic separation and thermal treatment, and met its corresponding limit value. The zinc content extracted by EDSE is as high as 58.6%, and the lead content is 5.5%.

EDR is the application of an electric field to the contaminated material, during which water splits at the anion exchange membrane and the suspension acidifies; the metal ions are electromigrated from the suspension to the electrolyte, and they then extracted to achieve the removal effect [74].

The purification level and efficiency of EDR depended on sediment properties, liquid-to-solid ratio, current density, the structure of the EDR device [74] and holding time. In addition, the experimental results of the stirred precipitated suspension were found to be significantly superior to the static setting [76]. Li and Kirkelund [77] applied pulsed stirring during the electrodialytic extraction of As, Cr, Pb, Cd, Cu and Zn from MSWI fly ash.

EDR can also be used as a pre-treatment method, such as deep removal of chloride ions from MSWI fly ash. Huang et al. [78] demonstrated that electrokinetic treatment effectively removes chloride from MSWI fly ash through targeted electromigration mechanisms. By applying an electric field, surface-layer Cl^−^ ions exhibit preferential migration toward the anode compared to those in deeper layers, with enhanced removal efficiency achieved through silicate additive optimization. The reaction equation for chlorine removal is as follows:

(3) $$\mathrm{Cathode}:\;2H_{2}O+2e^{-}\to 2{OH}^{-}+H_{2}↑$$

(4) $$\mathrm{Anode}:\;2H_{2}O-4e^{-}\to 4H^{+}+O_{2}↑$$

(5) $$\mathrm{Anode}:\;2{Cl}^{-}-2e^{-}\to {Cl}_{2}↑$$

Kirkelund et al. [79] demonstrated that incorporating electrodialytically treated MSWI fly ash into Greenland clay resulted in clay disks with minimized porosity and water absorption, suggesting their suitability as construction materials in cold climates.

#### 4.2.2. Chemical Extraction

Chemical extraction employs reagents to solubilize heavy metals from MSWI fly ash, facilitating their transfer into a leachable solution which is subsequently separated by adsorption. Extractants encompass inorganic acids (HNO_3_, HCl, H_2_SO_4_), organic acids (e.g., acetic, formic, oxalic), bases (NaOH, KOH), chelating agents (e.g., EDTA-2Na), and salts [80].

The leaching efficiency is markedly enhanced under acidic conditions, particularly for metal cations [81]. Different inorganic acids have different abilities in extracting heavy metals. HCI had the best extraction effect [25], which could extract most heavy metals. Extraction efficiency is highly dependent on the acid type: HCl achieves the most extensive removal; H_2_SO_4_and HNO_3_ display specific selectivity; H_2_SO_4_ extracts Cd and As more effectively, followed by Cu, Pb, and Zn; HNO_3_ favors Pb over Cd, Cu, and Zn [82]. Tang et al. [83] demonstrated that HCl is an effective leaching agent in a hydrometallurgical process for MSWI fly ash, achieving high leaching efficiencies of 100% for Cu, 75% for Zn, 100% for Cd, and 85% for Pb. Truong et al. [84] achieved the recovery of 99% As, 72–80% Cd, and 47–60% of Zn, Cu, Co, or Mn using H_2_SO_4_ as the leaching agent.

Among alkaline extractants, NaOH and KOH solutions show effectiveness for the immobilization and detoxification of hazardous heavy metals, as they facilitate the formation of stable, insoluble hydroxides. Kang et al. [85] studied that NaOH and KOH effectively leach heavy metals from MSWI fly ash and facilitate their fixation as carbonate precipitates via CO_2_ uptake, reducing leaching potential. In contrast, NH_4_OH shows superior leaching efficiency, especially for Zn and Cu, but inhibits precipitation due to ammonia complexation, complicating waste water treatment [86]. The complexation reactions of NH_3_ with Zn^2+^, Cu^2+^, Cd^2+^ are shown in Equations (6)–(9):

(6) $$NH_{4}^{+}+OH^{-}↔NH_{3}+H_{2}O$$

(7) $$4NH_{3}+{Cu}^{2+}↔Cu(NH_{3}{)}_{4}^{2+}$$

(8) $$4NH_{3}+{Zn}^{2+}↔Zn(NH_{3}{)}_{4}^{2+}$$

(9) $$4NH_{3}+{Cd}^{2+}↔Cd(NH_{3}{)}_{4}^{2+}$$

Chelating agents effectively extract heavy metals by forming soluble complexes through coordination, thereby facilitating metal leaching. In contrast to acid leaching, which often generates secondary pollution (e.g., acidic waste water and dissolved matrix components), chelating agents exhibit minimal interaction with the solid matrix while demonstrating high extraction efficiency and selectivity [87]. EDTA serves as an effective extractant for heavy metals from MSWI fly ash. Its leaching efficiency is influenced by dosage, leaching time, and the MSWI fly ash’s chemical properties [88]. Furthermore, such agents exhibit high selectivity; EDTA-2Na targets specific metals (e.g., Zn, Pb, Cu) and its performance is highly dependent on solution pH [89].

However, MSWI fly ash contains a large amount of alkaline metal oxides, which will greatly increase the use of acids and chelating agents. Therefore, it is necessary to carry out water washing pre-treatment before leaching. Furthermore, the overall cost of chemical leaching is substantially elevated by the complexity of heavy metal separation and the treatment of secondary waste streams. These economic constraints currently limit the widespread application of chemical extraction methods.

#### 4.2.3. Biological Extraction

**Figure 8:**
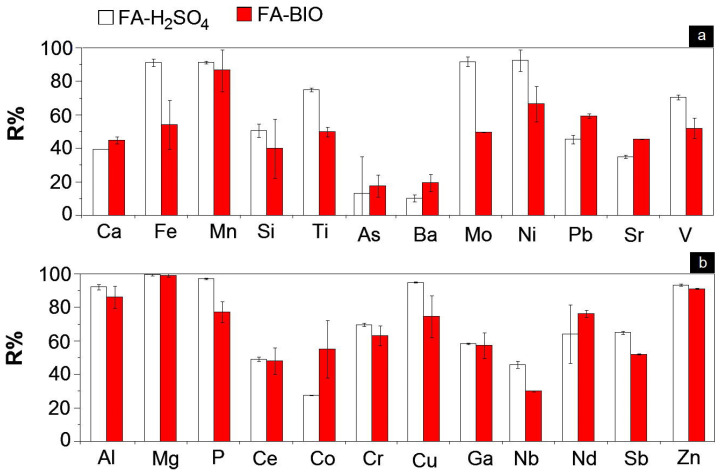
Comparison of leaching rate between H_2_SO_4_ leaching and bioleaching, (**a**) unvalued/hazardous elements; (**b**) critical/marketable elements [90].

Bioleaching mobilizes insoluble heavy metals from solid matrices into aqueous solutions via microbial activity or their metabolites, thereby converting them into soluble ionic forms amenable to recovery. Funari et al. [90] provided a comprehensive comparison between H_2_SO_4_ leaching and bioleaching (using a mixed culture of acidophilic bacteria) for metal removal from MSWI fly ash. The two methods demonstrated different efficiencies for various metal groups, highlighting a different metal selectivity. H_2_SO_4_ leaching achieved higher removal rate for Cu (95%), Fe (91%) and Ni (93%) in MSWI fly ash. Bioleaching, conversely, achieved superior removal rates for Nb (76%), Pb (53%) and Co (55%) (Figure 8).

The main bacteria used in biological leaching are as follows: *Leptospira ferrooxidans*, *Sulfurobacter*, *Thiobacillus*, *Acidophilus*, *Acidobacteria*, etc., among which *Thiobacillus ferrooxidans* [91], *Leptospira ferrooxidans* and *Thiobacillus thioxide* are the most widely used. These *Thiobacilus* are strictly aerobic chemoautotrophs, sensitive to dissolved low molecular weight organic matter, and can promote the extraction of heavy metals. Compared with the former, *Heterotrophic aspergillus niger* can produce various organic acids, which can dissolve metals effectively and have good potential.

Zhang et al. [92] used a two-stage biological leaching technology assisted by ferrous oxidation to treat mixed electroplating sludge with 12% slurry density in 24 h. Three microbial communities were screened, including sulfur-enriched microbial community, mixed-energy-enriched microbial community, and the ferrous-enriched microbial consortium. The mechanism of the leaching process is that H^+^ is mainly responsible for leaching acid soluble and reducible metals (such as most Cu, Zn, Ni), while Fe^3+^, as a strong oxidant, plays a key role in effectively attacking and converting stable residual metals, especially insoluble Cr. The passivation layer composed of iron/calcium compounds forming during the leaching process will hinder mass transfer.

The unique advantage of the ferrous-enriched microbial consortium lies in its ability to continuously regenerate H^+^ and Fe^3+^, thereby establishing and maintaining a high concentration gradient on both sides of the passivation layer, driving the leaching agent to penetrate the passivation layer and reach the reaction core. Among them, the ferrous-enriched microbial consortium had the best effect, with leaching rates of Cu, Ni, and Zn exceeding 96.11% within 24 h, and the Cr leaching rate reaching 75.4% [92].

Bioleaching is a more complex bio-hydrometallurgical process that involves a synergistic action where microorganisms produce H^+^ and regenerate powerful oxidizing agents like Fe^3+^. However, the technology faces several challenges for industrial application, including protracted leaching cycles, substantial cost for microbial cultivation, and inherent limitations in microbial tolerance to heavy metal toxicity.

### 4.3. Thermal Treatment

Detoxification of MSWI fly ash by thermal treatment means that organic pollutants (dioxins and furans, etc.) are degraded by heating, and heavy metals are firmly fixed in dense structures. According to the study, the different processing temperatures can be divided into the sintering method and melting/vitrification process. The choice of thermal treatment process depends on the characteristics of the product, not the process itself [93].

The chemical properties of the product after thermal treatment MSWI fly ash are stable, which can effectively prevent the migration of toxic substances; the reduction effect is significant, reaching over 50%; the heavy metals in MSWI fly ash can be recovered. In addition, stable slag can also be used as road substrate or other raw materials [94].

However, thermal treatment emits volatile metals (Pb, Zn, Cd) and PCDD/Fs; these can be captured by bag filters and activated carbon, producing secondary fly ash that must be retreated, which becomes an important factor limiting its promotion and application.

#### 4.3.1. Sintering

The sintering method refers to the recombination and densification of chemical phases of porous solid particles at temperatures below the melting point of their main components. The temperature of the process is generally controlled between 700 and 1200 °C [93]. During the treatment process, some harmful heavy metals (such as Cd, Pb, Hg, Zn, etc.) will evaporate, and dioxins will also decompose.

Yi et al. [95] showed the co-sintering of MSWI fly ash with iron ore to assess its impact on the behavior of PCDD/Fs. During the process, most PCDD/Fs from the added fly ash were decomposed under sintering temperatures exceeding 1000 °C, with degradation rates exceeding 75% for highly chlorinated congeners such as OCDD and HpCDD. However, increased chlorine input and extended sintering time, resulting from reduced bed permeability promoted the formation of lower- and mid-chlorinated PCDD/Fs. Consequently, although the total mass emission of PCDD/Fs decreased, the toxic equivalent emissions exceeded those from individual processes. According to the study’s findings, co-sintering may be a feasible option if advanced emission control measures are applied.

The positive relationship between process parameters (temperature and duration) and heavy metal volatilization, which poses environmental risks, is a major sintering challenge. This requires the development of procedures that ensure heavy metal safe treatment at reduced temperatures and with shorter sintering cycles. Zhou et al. [96] investigated the utilization of MSWI fly ash in cementitious materials through sintering (1050 °C for 180 min) and alkali activation. Sintering enhanced the pozzolanic activity of the MSWI fly ash by 81.7% and effectively stabilized heavy metals. The optimum mortar composition was found using response surface methodology based on a Box–Behnken design. It included a silicate modulus of 1.332, an alkali equivalent of 1%, 20% sintered MSWI fly ash, and 10.5% metakaolin. The flexural and compressive strengths of this optimized mixture were 8.5 MPa and 57.3 MPa, respectively, demonstrating noteworthy mechanical properties.

Before sintering, other pre-treatment steps are often required. Washing is an effective way to reduce the sintering temperature and time, which will reduce energy consumption, but requires additional waste water treatment [97].

The application of sintering for treating MSWI fly ash results in a consolidated product with reduced porosity and enhanced strength and density. This consolidation arises from solid phase transformations, recrystallization, and solid-state reactions, occasionally accompanied by limited liquid phase formation. Furthermore, a higher heating rate promotes denser crystallization, thereby improving mechanical properties. The resultant pore structure refinement effectively mitigates the leaching risk of hazardous substances, facilitating the safe resource utilization of the sintered product [98].

#### 4.3.2. Melting/Vitrification

Melting and vitrification are high-temperature treatments that involve converting raw materials, usually with the addition of glass-forming agents or other wastes, into a homogeneous liquid phase. This melt can turn into an amorphous phase, with controlled cooling, such as water quenching, a process known as vitrification. During the melting/vitrification process, hazardous components are effectively separated and immobilized within the resultant glass matrix [99]. The typical setting temperature ranges from 1200 to 1600 °C, depending on raw material composition.

During the melting/vitrification of MSWI fly ash, some of the low-boiling-point heavy metals (Cd, Zn, Pb) volatilize into the flue gas, while the others (Cr, Ni, Cu) are incorporated into the glassy slag matrix, where it is effectively immobilized, thereby significantly reducing leaching potential. The melting behavior and heavy metal migration of MSWI fly ash is strongly influenced by its chemical composition, particularly the content of Cl and S.

KCl and NaCl in MSWI fly ash commence volatilization at approximately 800 °C. Concurrently, they can undergo partial reaction with water vapor, leading to their conversion into HCl [100]. Notably, the presence of chlorides promotes the volatilization of specific metallic elements, such as Pb, which subsequently become enriched in the secondary fly ash collected from the flue gas treatment system. Yang et al. [100] indicated that the SiO_2_ and Al_2_O_3_ have a different effects on Cl behavior at 800–1400 °C. Both additives promote HCl release and lower the initial emission temperature. However, SiO_2_ significantly enhances Cl removal, achieving 99.72% removal at 1250 °C with 30% addition, by reacting with CaCl_2_ to form Ca-silicate and release HCl. In contrast, Al_2_O_3_ tends to retain Cl in residues by forming stable Cl-containing minerals like Ca_12_Al_10.6_Si_3.4_O_32_Cl_5.4_ at 1250 °C and 1400 °C, resulting in lower removal efficiency. In contrast, sulfates decompose over a broader temperature range, from approximately 600 °C to 1300 °C.

**Figure 9:**
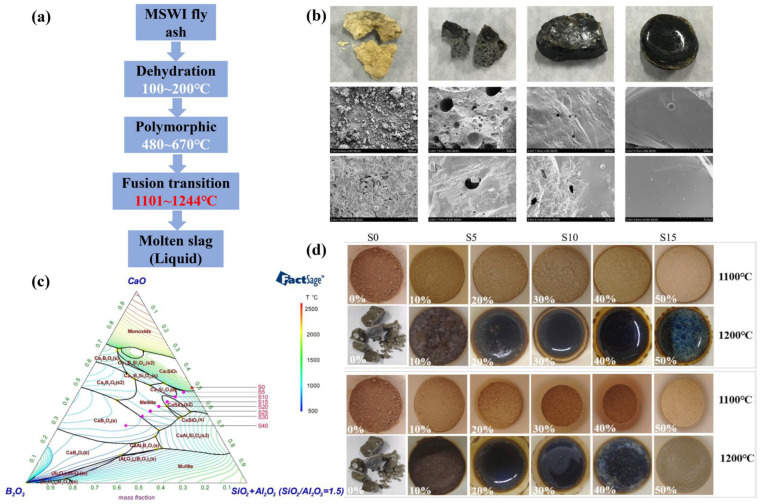
(**a**) Schematic of melting mechanism of MSWI fly ash [101]; (**b**) micrographs of glassy slag at 1150 °C [103]; (**c**) CaO-B_2_O_3_-SiO_2_/Al_2_O_3_ thermodynamic phase diagram [103]; (**d**) macroscopic of slag derived from different addition of MSWI fly ash [102].

The melting mechanism of MSWI fly ash is governed by phase transitions (Figure 9a). Thermodynamic drive dictates that the polymorph with the minimal Gibbs free energy is the most stable. Upon heating, this stability is disrupted, triggering a phase transition and altering the equilibrium state of the system [101].

The mixture composition is a key determinant of the thermal requirements for MSWI fly ash melting/vitrification, as it directly influences energy consumption and the target processing temperature. The formation of a low-melting-point eutectic, thermodynamically favored by these interactions, counteracts the effect of high-melting-point compositions (e.g., CaO (2572 °C); SiO_2_ (1723 °C); Al_2_O_3_ (2054 °C)), thereby depressing the system’s fusion temperature [102]. Gao et al. [103] achieved a significant reduction in the melting temperature of MSWI fly ash by using B_2_O_3_ as a fluxing agent in the CaO-B_2_O_3_-SiO_2_/Al_2_O_3_ system, which lowered the melting temperature from 1211 °C to 986 °C (a reduction of 18.6%) with a 15 wt% B_2_O_3_ addition (Figure 9b,c). Zhao et al. [102] presented that co-melting MSWI fly ash with biomass ashes (straw ash and rice husk ash) can significantly reduce the melting temperature and facilitate efficient vitrification. Optimal additions of 20–50% straw ash and 20–40% rice husk ash lowered the melting point of MSWI fly ash by approximately 100 °C, achieving minimums of 1182 °C and 1184 °C, respectively. Vitrification was complete at 1200 °C when the liquid phase content exceeded 50%, resulting in a stable, glassy slag (Figure 9d).

Vitrification has many advantages, such as the effective fixation of heavy metals, the ability to accommodate various forms of MSW, high maturity, significant capacity reduction effects, and the recyclability of products. However, the main drawback of vitrification is the cost incurred due to energy consumption (1t MSWI fly ash is about 600–1000 kWh) during the process [99,103].

## 5. MSWI Fly Ash Resource Utilization

After detoxification, MSWI fly ash has two primary disposal options: landfilling or resource recovery. Recycling-treated MSWI fly ash is a raw material that offers the dual benefit of reducing production costs and minimizing environmental risks, with long-term product stability being a key consideration. This section explores resource utilization pathways, including ceramic bricks, glass ceramics, glass ceramic foams, road substrates, cement and concrete.

From a spatial perspective, landfill is still the main method for the disposing of MSWI fly ash globally. However, China, Japan, and South Korea are trying low-carbon technologies to treat MSWI fly ash and reduce land resource consumption. From the perspective of time, the recycling process of MSWI fly ash mainly focuses on the preparation of simple building materials from 2010 to 2015, focusing on the preparation of high value-added glass ceramics/foam from 2016 to 2020, and emphasizing the trinity of “harmless, resourceful and low-carbon” from 2021 to 2025.

### 5.1. Ceramic Bricks

Traditional ceramic bricks usually come from clay, limestone, and coal fly ash. However, an assessment of sustainable alternatives is required due to the limited availability and comparatively low economic worth of these typical resources. As a result, selecting MSWI fly ash as an essential raw material for ceramic brick manufacturing gives a promising option for further research. Xu et al. [104] developed a novel method for utilizing MSWI fly ash in fired bricks by pelleting it with waste glass powder before sintering at 950–1050 °C. The process significantly enhanced brick compressive strength (>25 MPa), reducing chloride volatilization by 31.09–48.11% and leaching by 18.13–30.99%, while increasing chloride solidification efficiency to over 93%.

However, as the raw material of the main production, toxic substances contained in MSWI fly ash have the potential to harm animals, plants, and even humans, based on whether the biological toxicity of MSWI fly ash is retained by ceramic bricks. Kirkelund et al. [79] examined the incorporation of electrodialytically treated MSWI fly ash as a partial clay replacement in brick manufacturing. The electrodialytic pre-treatment effectively removed soluble salts (e.g., NaCl, KCl) and reduced the total content of heavy metal, although it increased the leaching of Cr and Zn. Bricks incorporating 10–30% treated MSWI fly ash were sintered at 1000 °C for 24 h, showing that bricks made with treated MSWI fly ash exhibited lower porosity and water absorption, which is beneficial for cold climates, though mechanical properties generally decreased with higher MSWI fly ash content. Deng et al. [105] designed an experiment to evaluate the biological toxicity of MSWI fly ash-based ceramic bricks using bacteria (*Escherichia coli* and *Staphylococcus aureus*) and mammalian cells (MG63). The results showed that the heavy metal leaching concentration of MSWI fly ash-based ceramic bricks was lower than the limit value specified in GB16889-2008. Moreover, MSWI fly ash-based ceramic bricks have good biosafety to the aforementioned organisms. This experimental result will promote the further utilization of MSWI fly ash-based ceramic bricks.

### 5.2. Glass Ceramics

Glass ceramics are polycrystalline materials produced through the controlled crystallization of a base glass of specific composition, with or without nucleating agents. Glass ceramics refers to a specific composition of basic glass with or without nucleating agents, which undergoes crystallization thermal treatment at a certain temperature system, uniformly precipitating a large number of small crystals inside the glass, forming a dense multi-phase composite of microcrystalline and glass phases. Glass ceramics is a new type of comprehensive glass for building materials, which has the dual characteristics of glass and ceramics.

The fabrication of glass ceramics—the most technologically mature waste-derived glass product—consists of the incorporation of binders which enables the production of glass ceramics with properties comparable to those of traditional ceramics, but at significantly lower sintering costs [106]. The preparation of glass ceramics from MSWI fly ash can be combined with melting/vitrification, and the formed glass melt can be directly nucleated and crystallized to obtain glass ceramics.

The glass ceramics prepared from MSWI fly ash has high mechanical strength, hardness, significant corrosion resistance, and can also be insulated; it is sound-absorbing, fireproof, moisture-proof, etc. Ordinary glass atoms are arranged irregularly, making it extremely fragile, while glass ceramics is composed of crystals with regular atomic arrangements, making it more ductile than ceramics. A comprehensive understanding of the crystallization mechanism, involving nucleating agents, kinetics, glass network transformation and the immobilization mechanism of heavy metals is pivotal for advancing the clean production of glass ceramics derived from MSWI fly ash [107]. Consequently, these areas constitute the current research priorities toward developing sustainable and energy-efficient valorization strategies. Ding et al. [108] found that as the average cooling rate decreases, the starting temperature and ending temperature of crystallization gradually increase. When the average cooling rate decreases from a higher value to 1.78 °C/s, the temperature range of the main crystallization zone changes from 1362.4 to 1311.3 °C to 1372.7–1348.6 °C. This means that under slower cooling, crystals begin to precipitate at higher temperatures and end their growth at higher temperatures.

Li et al. [109] successfully incorporated up to 70% MSWI fly ash with synergistic chromium slag solidification. They reported that increasing the MSWI fly ash ratio enhanced basicity, promoting gehlenite formation while inhibiting anorthite and diopside ferrous. A 50% MSWI fly ash blend demonstrated optimal properties (bulk density: 2.78 g/cm^3^, compressive strength: 226.85 MPa, water absorption: 0.64%). In contrast, performance was severely compromised at a 70% blending ratio of MSWI fly ash, with compressive strength dropping to 39.07 MPa and water absorption rising to 1.97%, attributed to excessive gehlenite increasing viscosity and porosity. In addition, while 10% chromium slag promoted crystallization, 30% addition resulted in multi-phase accumulation, porosity, and decreased density and mechanical strength.

A glass ceramic from a mixture of MSWI FA, secondary aluminum ash, and fluorite tailings was developed by Zhang et al. [110]. By adjusting the composition within the CaO-SiO_2_-Al_2_O_3_ system, the melting point was significantly reduced from 2196.4 °C to approximately 1200 °C. Raman spectroscopy was used to analyze the structural evolution of [SiO_4_] tetrahedra in glass networks, and five structural units, Q^0^–Q^4^, were identified through Gaussian fitting. As the Al_2_O_3_ content increased, Q^2^ decreased and Q^3^ increased in the glass network. Q^2^ and Q^3^ unit, respectively, dominated the depolymerization and polymerization of the glass network, resulting in a decrease in the non-bridging oxygen/tetrahedral ratio from 1.90 to 1.87, which signifies enhanced network polymerization. The optimized glass ceramic exhibited a peak Vickers hardness of 8.71 GPa. Furthermore, it demonstrated excellent heavy metal stabilization, with solidification efficiencies of 99.82% for Ba, 98.51% for Cu, and 99.12% for Cr. Due to the presence of Cl, heavy metals such as Zn, Pb, Cd, and Cu were efficiently removed via chlorination–volatilization, with the highest rates reaching 97.47%, 99.18%, 100%, and 45.96%, respectively.

**Figure 10:**
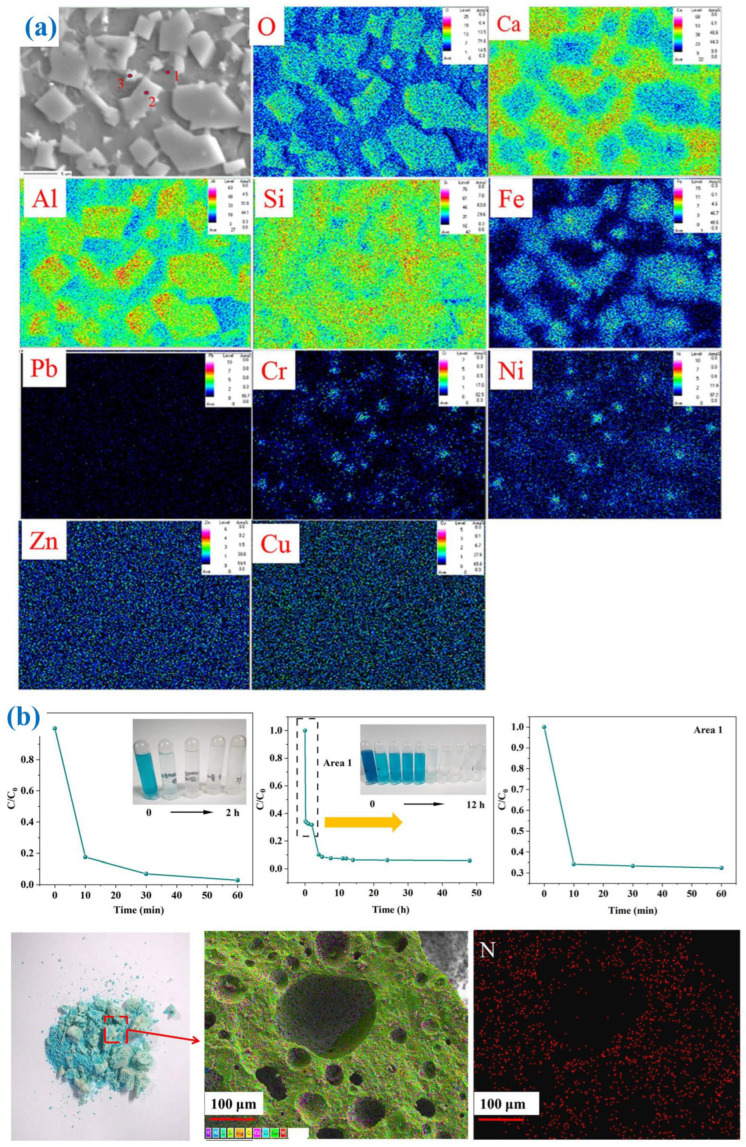
(**a**) Mapping of glass ceramics with heavy metals [112]; (**b**) glass ceramic foams derived from MSWI fly ash [118].

The development of glass ceramics using MSWI fly ash, pickling sludge and waste glass was evaluated, with a focus on the solidification mechanisms of heavy metals and the feasibility of a one-step sintering process [111,112]. It was found that Cr and Ni were effectively immobilized within spinel-structured crystals via solid solution formation, whereas Zn, Cu, and Pb were physically encapsulated in the glass matrix (Figure 10a). After extraction by TCLP, the prepared glass ceramics meets the TCLP toxicity threshold and provides a promising route for the high-value recycling of hazardous solid wastes [111,112]. The chemical forms of heavy metals were analyzed using the BCR three-step continuous extraction method, which can be divided into acid soluble, reducible, oxidizable, and residual states. The first three are in the migratory state, and the residual state determines environmental stability. In the glass obtained by melting, highly toxic Cr is mainly in an oxidizable and reducible state, while Ni contains 10% acid soluble state, which poses a high risk of migration; in the crystallized glass ceramics, the residual state of Cr and Ni accounts for 91%, while the acid soluble state is significantly reduced. There is no significant difference in the forms of Zn and Pb between glass and glass ceramics, while the proportion of reducible states of Cu in glass ceramics increases with the increase in content [112]. Crystallization treatment promotes the transformation of heavy metals from a migratory state to a residual state, significantly enhancing their environmental stability.

### 5.3. Glass Ceramic Foams

The synthesis of glass ceramic foams from MSWI fly ash is conventionally achieved via three principal methods: powder sintering, alkali-activated foaming, and inorganic gel casting [113]. The most widely adopted method, powder sintering, encompasses a series of steps including the homogenization of MSWI fly ash (or vitrified MSWI fly ash) with supplementary raw materials and foaming agents (e.g., C, CaCO_3_, SiC), subsequent ball milling and shaping, and final consolidation through thermal treatment to form the foamed microstructure [114].

Barracco et al. [115] developed glass ceramic foams from MSWI fly ash via vitrification and thermal treatment, comparing the environmental impacts of different silicon sources (bottom ash, waste glass and silica sand) and heat recovery methods through life cycle assessment. CaCO_3_ was used as the foaming agent, decomposing at 800 °C to release CO_2_ and generate pores. The vitrification temperature ranged from 1300 to 1450 °C, depending on the silicon source. Results indicated that using bottom ash as the silicon source combined with methane-assisted heat recovery was the most environmentally sustainable option, yielding only 0.467 kg CO_2_-eq emissions and 9.26 MJ energy consumption per kilogram of glass ceramic foams, while effectively immobilizing heavy metals.

Successful foaming in the powder sintering method requires temperatures above the material’s softening point to establish a viscous liquid phase for gas entrapment. Within this sintering window, elevated temperatures and extended times lower the melt viscosity and accelerate gas release, resulting in bubble coalescence and foam coarsening [116]. Therefore, glass composition, foaming temperature, foaming time, foaming agent addition, etc., are all factors that need to be adjusted. However, the viscosity, crystallization, and surface tension of the melt at high temperatures also have an impact on the formation of porous structures [113]. These three factors also affect each other in high-temperature environments, such as the intensification of crystallization leading to an increase in melt viscosity. The coupling regulation between these three factors is similar to a black box reaction, making quantitative control challenging. To address this, alternative strategies like inorganic gel casting and mechanically alkali-activation foaming methods have been proposed for more controllable and predictable pore structure regulation [117].

Prior work has successfully developed glass ceramic foams using two distinct solid waste upcycling strategies [118]. One involved the co-treatment of MSWI fly ash and secondary aluminum dross via melting for metal recovery, followed by particle-stabilized foaming of the vitrified slag [119]. Another approach utilized alkali-activation foaming to convert vitrified MSWI fly ash and bottom ash into glass ceramic foams with hierarchical pores, exhibiting high porosity (77.22–82.94%) and low bulk density (0.68–0.91 g/cm^3^) [120]. The obtained glass ceramic foams with hierarchical pores can be used as an adsorbent (Figure 10b) [118].

### 5.4. Road Substrates

**Figure 11:**
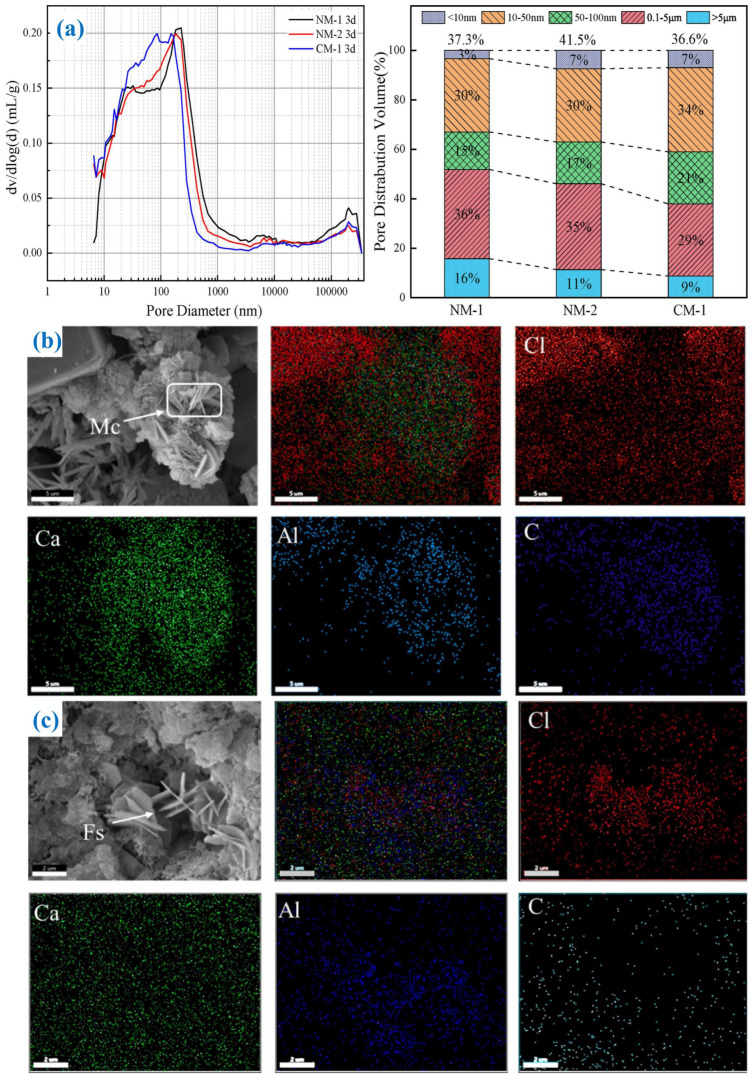
(**a**) Pore size of cement derived from MSWI fly ash after carbon curing [123]; SEM and EDS of carbonate phases (**b**) 3d; (**c**) 28d [123].

The valorization of MSWI fly ash in asphalt pavements has been explored as a replacement for conventional limestone mineral filler in asphalt mortar (Figure 11a). Asphalt is a highly hydrophobic material that can form a barrier to prevent any pollutants from seeping into the environment from MSW [6]. Asphalt mortar, which constitutes a mixture of mineral filler and asphalt binder, serves as the binding matrix for aggregates in asphalt mixtures, and its performance fundamentally governs the overall properties of the composite.

Yan et al. [6] conducted a performance comparison between asphalt mortar, incorporating MSWI fly ash and conventional limestone mineral filler. Because of its advantageous physical properties, such as its tiny particles, consistent porosity, large surface area, and active transition metal species, they determined that MSWI fly ash was an excellent substitute filler. By increasing durability and decreasing fatigue-related defects like cracking, Tang et al. [121] showed that adding a layer of solidified MSWI fly ash as a structural interface between the unbound base and roadbed significantly enhances pavement performance.

### 5.5. Cement and Concrete

MSWI fly ash has a mineral composition similar to cement, which can replace some cement or aggregates and reduce natural resource consumption, but it often requires pre-treatment techniques such as water washing, carbonization, or vitrification to achieve high fly ash addition [122].

Zhao et al. [123] treated MSWI fly ash and cement paste with carbon stirring and curing, concentrating on effects on mechanical properties as well as the solidification effect of chloride ions and heavy metals. The results showed that employing 1.5 min of conventional stirring followed by 1.5 min of carbon stirring resulted in a compressive strength of 19.29 MPa at 28 days for cement slurry incorporating 60% MSWI fly ash. Carbon stirring promotes the production of CaCO_3_ and monocarbonate, optimizes pore structure, increases the solidification of Cl^−^ and heavy metals, and has a better effect than traditional carbon curing (Figure 11a–c). The feasibility of utilizing a dosage of 25% of MSWI fly ash as a partial substitute for cement was investigated by Yan et al. [124]. This mixture not only successfully immobilized heavy metals, mitigating their leaching potential, but also contributed to superior material performance, including enhanced crack resistance.

Although businesses can already dispose of MSWI fly ash, variable product quality has resulted from a lack of standardized norms, which impacts the engineering durability, safety, and quality of concrete. The MSWI fly ash used in cement and concrete must have certain characteristics such as activity and low water demand ratio. Standards for MSWI fly ash used in cement and concrete must be developed immediately in order to standardize the application of MSWI fly ash in these materials.

## 6. Conclusions, Prospect and Limitations

This review provides the mechanisms, advantages, limitations and potential improvements in MSWI fly ash treatment. Key findings include the following:

(1) Stabilization, extraction, etc., have proved to be effective in realizing the solidification of heavy metals and the removal of dioxins in MSWI fly ash. However, there are a number of challenges with these technologies, including high costs, excessive energy use, inadequate scale, and secondary pollutants. Combining chemical stabilization with cement solidification enhances heavy metal long-term stability and reduces environmental hazards. Cement solidification emits ~0.9 t CO_2_/t, geopolymers offer lower CO_2_ footprint and better immobilization (e.g., Pb > 99.6%), but ash component variability hinders standardization.
(2) Electrodialytic methods achieve high removal of Cd (98%) but it is limited for Pb (12%). However, its application to MSWI fly ash leachates is hampered by membrane fouling caused by colloidal silicates and aluminates. Bioleaching is promising for critical metals (e.g., Zn, Pb, Ni) but slow.
(3) Sintering and vitrification degrade dioxins (>99%) and stabilize heavy metals, but energy consumption is high (600–1000 kWh/t), which has become an important factor limiting its application. The chemical properties of the glass products are stable and the volume reduction effect is remarkable. Pre-treatment before sintering and adding fluxing agents to lower the melting point of the system during melting/vitrification are effective measures to improve economic feasibility.
(4) MSWI fly ash serves as a raw material for ceramic bricks, glass ceramics, glass ceramic foams, road substrates, and concrete, but low-carbon, cost-effective integrated processes and long-term environmental stability needs further research. The conversion into ceramic bricks achieves dual goals of heavy metal stabilization and reuse. Incorporating MSWI fly ash into asphalt or concrete also improves crack resistance and service performance.

This review has several limitations. First, the literature screening focuses on core literature in both Chinese and English, with insufficient inclusion of non-English. Second, there is limited research data on some emerging technologies, such as microbial treatment, and it is currently impossible to conduct systematic quantitative comparisons. Third, the long-term field performance data are scarce. Finally, the review focused on technical aspects; economic and policy analyses were not systematically covered. Existing research mainly focuses on laboratory-scale experiments, and there is a scarcity of unified and accurate commercial application data (e.g., differences in regional energy prices, equipment investment, and operation and maintenance costs lead to large variations in actual treatment costs).

## Figures and Tables

**Table 1:** Disposal methods for MSWI fly ash in different countries [14].

Process Mode	Country	Disposal Method
Secured landfill	USA	Co-disposed with bottom ash in MSWI residue-exclusive landfills
	Canada	Disposal in hazardous waste landfills after treatment
	Germany	Disposal in underground sites like abandoned salt mines
Solidified and stabilized before landfill	France	Disposal by hydraulic binders (such as cement, lime, furnace slag, etc.) and then stored in specific landfills
	China	
	Italy	
Resource utilized before landfill	Japan	Pretreated by melting and used in road construction

**Table 2:** Existing form of heavy metals and their sources in MSW [20].

Heavy Metals	Important Source	Existing Form
Hg	Residues from caustic soda production processes, pigments from plastics, thermometers, electronic components, and batteries	HgCl_2_
Cr	Stainless steel, dye, paint	Cr_2_O_3_, CrO_3_
Zn	Galvanized materials, electronic products, paper, environmental drugs	ZnO, ZnCl_2_, 2ZnCO_3_·3Zn(OH)_2_
Cd	Coatings, batteries, stabilizers/softeners	CdO, CdCl_2_, Cd(OH)_2_
Pb	Pigments, plastics and batteries and some alloys	PbCl_2_, PbO, PbCO_3_
Ni	Stainless steel, nickel battery, etc.	NiO

**Table 3:** Leaching characteristics of heavy metals from MSWI fly ash by different chemical stabilizers [14,59].

Chemical Agent	Cd		Cr		Cu		Ni		Pb		Zn	
	N	A	N	A	N	A	N	A	N	A	N	A
Phosphate	-	-	+	-	-	+	-	+	-	+	-	-
Silicate	/	/	/	/	-	-	/	/	-	-	-	-
Sulfide	-	/	+	/	+	/	-	/	-	+	-	/
Iron oxide	-	-	+	/	-	/	/	/	-	-	-	-
Thiourea	-	/	-	/	/	/	/	/	-	/	/	/
Dithiocarbamate	-	-	+	+	-	-	-	+	-	+	-	+
Dithiocarbamic acid dipotassium	-	/	/	/	/	/	-	/	-	/	/	/
Thiourea	-	/	/	/	/	/	-	/	-	/	/	/

“-” in the table indicates that the leaching capacity is inhibited; “+” indicates that the leaching capacity is improved; “/” indicates that the leaching capacity has not been studied. “N” represents neutral conditions for leaching capacity, and “A” represents acidic conditions for leaching capacity.

**Table 4:** Summary of the solidification/stabilization style and efficiency.

S/S Style	Addition Materials	MSWI Fly Ash Content	Major S/S Product	Heavy Metals S/S Efficiency	Ref.
Cement	Sulfoaluminate cement, sand	0–20%	Friedel’s salt, anhydrite	100% retention efficiency for Hg, Cu, Zn, Pb, and Cd	[61]
	CaO, MgO, blast furnace slag	50–90 wt.%	Calcium silicate hydrate, magnesium silica hydrate	99.8% for Zn and 99.7% for Pb	[62]
	42.5 Portland cement	20%	Quartz, calcite, ettringite and hydrocalumit	Cd, Cr, Pb, Se, Zn and Ba met Chinese standard	[56]
Geopolymer	RM, blast furnace slag	0–52.5 wt.%	Calcium aluminosilicate hydrate, Friedel’s salt, ettringite	99.6% for Pb	[49]
	Metakaolin, NaOH, sodium silicate	70%	Quartz, gehlenite, gypsum	89% for Cr, >92% for the others heavy metals	[63]
	Coal gangue, NaOH, sodium silicate	30–60%	Quartz, gismodine, calcite, portlandite	Cu and Zn can be solidified but stabilization ability of Cd and Pb was limited.	[64]
	RM, NaOH, sodium silicate	20–80%	Sodium aluminosilicate, pyrophyllite, calcite,	Cd, Cr, Cu, Ni, Pb and Zn met the USEPA limits	[65]
	Liquid water glass, sodium hydroxide, slag, coal gangue, fly ash	20–40%	Calcite, quartz, hydrocalumite, ettringite,	Maximum curing rates of Cd, Pb and Cr were 80.97%,87.90% and 76.68%	[53]
	Blast furnace slag, construction and demolition waste, Na_2_O	20%	Quartz, calcite, calcium silicate hydrate, calcium aluminosilicate hydrate,	Cd, Cr, Pb, Se, Zn and Ba met Chinese standard	[56]
Chemical	Oligomeric dithiocarbamate	66.6%	CaCO_3_, CaSiO_3_, NaCl, KCl	Cd, Pb and Zn met landfill standards in China	[66]
	Sodium diethyldithiocarbamate, phosphate	<50 wt.%	NaCl, KCl, CaCO_3_, CaSO_4_, CaSi_2_O_5_	Phosphate can decrease the leaching of Zn, Cd and Cr	[67]

## Data Availability

No new data were created or analyzed in this study. Data sharing is not applicable to this article.

## Abbreviations

The following abbreviations are used in this manuscript:

C-A-H	Calcium aluminate hydrate
C-A-S-H	Calcium aluminosilicate hydrate
C-S-H	Calcium silicate hydrate
DC	Direct current
EDSE	Electrodialytic separation
EDR	Electrodialytic remediation
EDTA	Ethylenediaminetetraacetic acid
EU	European Union
I-TEQ	International toxic equivalent
MSW	Municipal solid waste
MSWI	Municipal solid waste incineration
PCDD	Poly-chlorinated dibenzo-p-dioxin
PCDF	Poly-chlorinated dibenzofuran
PIC	Products of incomplete combustion
S/S	Solidification/stabilization
SEM	Scanning electron microscopy
TCLP	Toxicity characteristic leaching procedure
XRD	X-ray diffraction
